# Structural, electronic, optical and photocatalytic properties of KTaO_3_ with NiO cocatalyst modification

**DOI:** 10.1039/d2ra06425a

**Published:** 2022-11-10

**Authors:** Bin Wu, Jinzhen Cai, Xin Zhou

**Affiliations:** Organ Transplant Center, Fujian Medical University Union Hospital Fuzhou 350000 China caijinzhen@sina.com; College of Environment and Chemical Engineering, Dalian University Liaoning 116622 China zhouxin@dlu.edu.cn

## Abstract

KTaO_3_ loaded with NiO cocatalyst is an efficient photocatalyst that has been widely applied to various photocatalytic reactions. In this work, density functional theory calculations have been utilized to investigate the interfacial geometries, electronic structures, charge transfer, optical absorption, and water oxidation mechanism of the NiO(001)/KTaO_3_(001) slab model. The formation of O–Ni and Ta–O interfacial bonds is thermodynamically stable, indicating a covalent interaction between the two components of the heterostructure. The calculated density of states using the PBE+U and HSE06 methods shows that in the NiO/KTaO_3_ heterostructure, the valence band maximum and conduction band minimum of NiO are located above those of KTaO_3_, indicating the formation of type-II band alignment. Upon light irradiation, the photogenerated electrons accumulate at the KTaO_3_ side and photogenerated holes gather at the NiO side. The difference in electrostatic potentials around the interface as a driving force boosts the migration of electrons and holes to different domains of the interface, which is beneficial to extending the lifetime of photoinduced carriers and improving the photocatalytic activity of the KTaO_3_ system. The formation of an interface between NiO and KTaO_3_ evidently reduces the overpotential of the oxygen evolution reaction because the adsorption of intermediates in the water oxidation process becomes more moderate. Our results provide new insights into understanding the influence of loading NiO cocatalyst on the photocatalytic performance of KTaO_3_, which provides a theoretical guidance for designing new semiconductor-based photocatalysts.

## Abstract 2

Density functional theory calculations reveal that NiO serves as an oxidation cocatalyst to form type-II band alignment with KTaO_3_, which suppresses the recombination of photoinduced carriers and enhances the photocatalytic activity of KTaO_3_.

## Graphical abstract

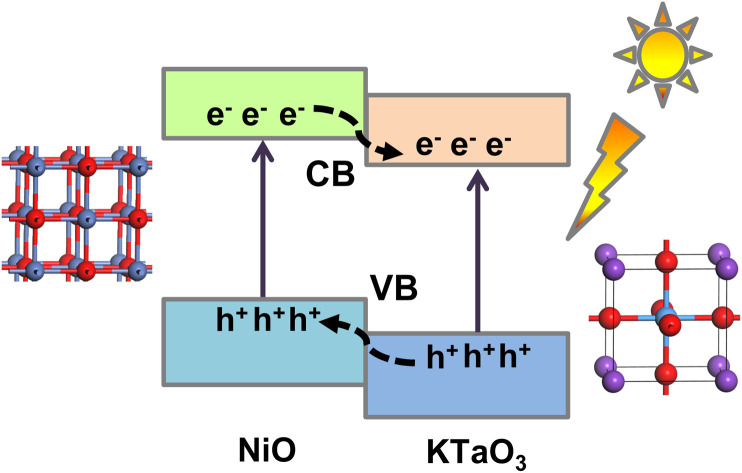

## 1. Introduction

Perovskite-type oxides are a category of semiconductors having the common formula ABO_3_, in which an A cation with a larger ionic radius is twelve-coordinated to oxygen atoms and a B cation with a smaller ionic radius is six-coordinated to oxygen atoms.^1^ Since the A and B sites can be occupied by most of the metal elements in the periodic table, the rational combination of different metal ions extends the family of perovskite oxides.^2^

Due to their simple, flexible and stable structures, perovskite oxides have been applied to a variety of fields, such as photovoltaics,^3^ photocatalysis,^4–6^ optoelectronics and ferroelectrics.^7,8^ In varied experimental conditions, ABO_3_ usually undergoes lattice distortion to varying degrees, leading to the transformation of crystal phases into low symmetry structures.^9–12^ As a representative perovskite oxide, KTaO_3_ has a nearly ideal cubic structure at room temperature,^13,14^ which has currently attracted considerable attention as a highly efficient photocatalyst in hydrogen evolution,^15–18^ pollutant degradation,^19–22^ and CO_2_ reduction.^23,24^

Semiconductor-based photocatalysis has recently become one of important strategies for solving global energy and environmental issues.^25–27^ A particulate photocatalytic system generally consists of semiconductors and cocatalysts. Semiconductors are responsible for absorbing solar light to generate the photoinduced electrons and holes. Cocatalysts loaded on the surface of semiconductors are considered to be active sites of photocatalytic reactions to benefit the separation of carrier charges. The type, size and structure of cocatalysts are important factors in controlling the reaction activity of a photocatalytic system.^28–31^ In terms of KTaO_3_ photocatalytic system, NiO was found to be the most efficient cocatalyst, which is also widely utilized in other photocatalytic materials.^32–38^ Kato and Kudo investigated the effect of loading cocatalysts on the photocatalytic performance of ATaO_3_ (A = Li, Na and K).^39^ It was found that the photocatalytic activity of KTaO_3_ for overall water splitting was improved after loading a NiO cocatalyst, which was attributed to the suitable conduction band level composed of Ta 5d orbitals and the delocalization resulted from the proper distortion of TaO_6_ octahedra. Ishihara *et al.* reported that Zr-doped KTaO_3_ loaded by NiO exhibited larger formation rate of H_2_ by photo-decomposing water than that of a famous photocatalyst Pt/TiO_2_ under the same reaction conditions.^40^ Shao *et al.* found that loading a small amount of NiO as the cocatalyst resulted in the notable enhancement of reaction activity of KTaO_3_ in photocatalytic reduction of CO_2_ to methanol.^24^ The highest yield reached when 2 wt% NiO was added on KTaO_3_.

Although experimental observations have confirmed the positive effect of loading NiO on enhancing the photocatalytic performance of KTaO_3_, the function of NiO cocatalyst in the photocatalytic system is not well understood since the details of the interface are quite difficult to obtain from experimental techniques. In this respect, first-principles density functional theory (DFT) calculations can be useful, which have been extensively applied in studying structural, electronic and optical properties of bulk, surfaces and interfacial structures of KTaO_3_.^41–47^ Theoretical results have provided reasonable explanations and reliable predictions on experiments. In this work, we have constructed a NiO/KTaO_3_ slab model and performed DFT computations to study the structural details and stability of the interfacial structure, to investigate the electronic and optical properties, to explore the carrier migration at interface, and to reveal the mechanism of the enhancement of photocatalytic activity by loading NiO cocatalyst on KTaO_3_ surface.

## 2. Computational details

Spin-polarized calculations have been performed by means of the projector augmented wave method,^48,49^ as implemented in the plane-wave basis code Vienna *Ab initio* Simulation Package (VASP).^50,51^ The exchange–correlation potentials are represented by the Perdew–Burke–Ernzerhof (PBE) functional within the generalized gradient approximation (GGA).^52,53^ The plane-wave energy cutoff is set to 400 eV and the Brillouin zone are sampled with Monkhorst–Pack meshes of 9 × 9 × 9 for bulk NiO and KTaO_3_, 9 × 9 × 1 for the NiO(001), KTaO_3_(001), and NiO(001)/KTaO_3_(001) heterostructure, respectively. PBE+U method has been applied to calculate electronic structures due to the strong correlation of 3d electrons of Ni ions.^54^ The onsite parameter *U*_eff_ (= *U* − *J*) is set to be 5.3 eV for Ni 3d electrons, which was calculated self-consistently by Ferrari *et al.*,^55^ in the range of 4.6–6 eV interval found in the previous literatures,^56–58^ and applied to study NiO(001) surface in the recent works.^59,60^ The ground state of NiO is antiferromagnetic spin ordering and the PBE+U local magnetic moment on the Ni ion is 1.66 *μ*_B_,^58,59^ which is consistent with the experimental value of 1.64 *μ*_B_ and the previous calculated results of 1.65 *μ*_B_ and 1.68 *μ*_B_.^59,61,62^ The DFT-D3 method is applied to describe the internal interactions in interfacial structures.^63–65^ Structural relaxations are terminated until the force on each atom is less than 0.01 eV Å^−1^ and the electronic energy is converged to 10^−5^ eV. We also employ a HSE06 hybrid density functional approach with a screening parameter *μ* of 0.2 Å^−1^ and a *α* value of 0.25 to make a comparison study.^66–68^

## 3. Results and discussion

### 3.1. Geometry and stability

**Fig. 1:**
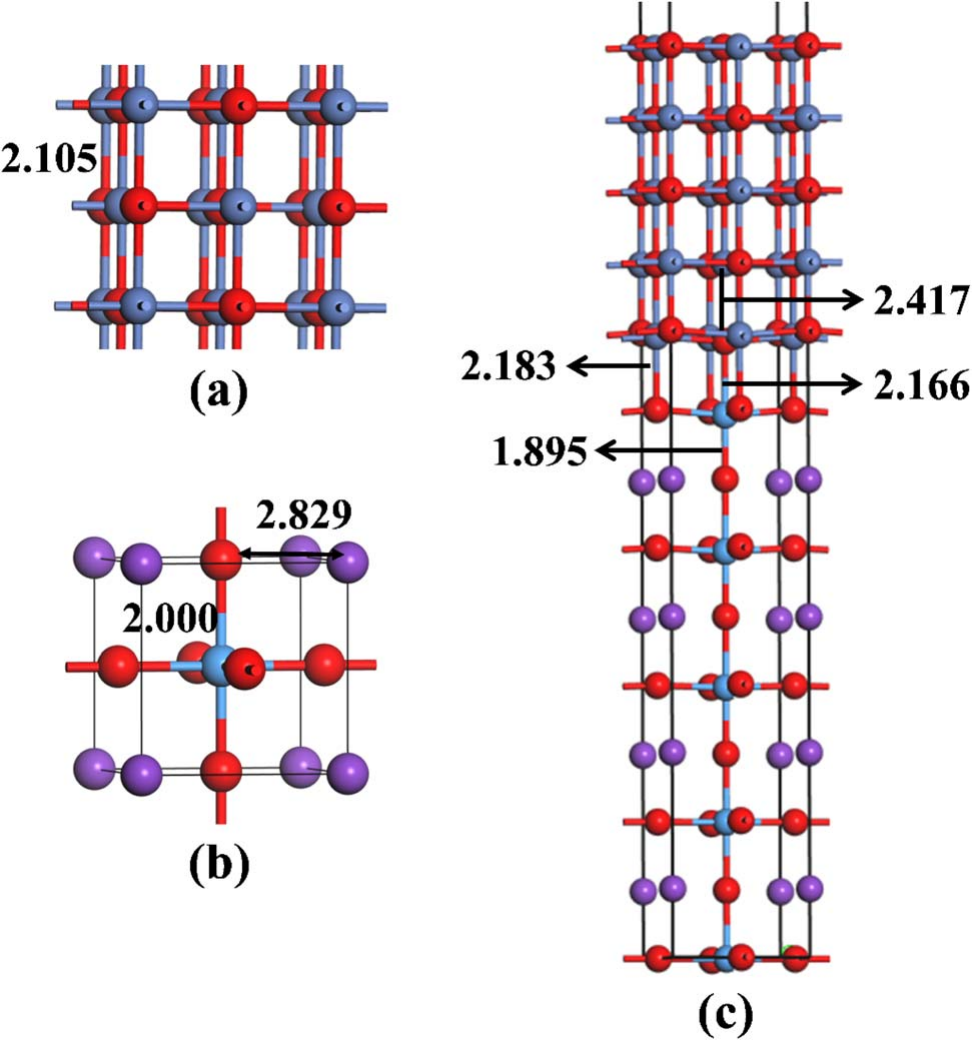
Optimized structures of (a) bulk NiO, (b) bulk KTaO_3_, and (c) NiO(001)/KTaO_3_(001) interface with key bond lengths. The red, blue, purple and azure balls represent O, Ni, K and Ta atoms, respectively.

Before building NiO/KTaO_3_ interfacial structure, fully optimizations are carried out for bulk phases. According to previous investigations,^41,69^ the cubic phase of KTaO_3_ with a space group of *Pm*3̄*m* is adopted in the present work. The crystal structures of bulk NiO and KTaO_3_ are shown in Fig. 1(a) and (b), respectively. The relaxed lattice constants of unit cells are *a* = *b* = *c* = 4.209 Å for NiO and *a* = *b* = *c* = 4.001 Å for KTaO_3_, which are in good agreement with previous experimental and theoretical reports.^61,70,71^ NiO has a NaCl-type structure and the optimized bond length of Ni–O is 2.105 Å. Bulk KTaO_3_ has a cubic structure, in which the relaxed distances are 2.000 Å for Ta–O bond and 2.829 Å between the nearest K and O atom.

Our model representing the NiO/KTaO_3_ interface is presented in Fig. 1(c), which is composed of a 1 × 1 NiO(001) slab with five alternating atomic layers and a 1 × 1 mirror-symmetric TaO_2_-terminated KTaO_3_(001) slab including nine atomic layers.^44,72^ The calculated lattice constants of bulk KTaO_3_ and NiO are applied for the lattice matching. This gives rise to a 5% lattice mismatch. The vacuum thickness is more than 15 Å, which is sufficient to avoid the interaction between periodic images. After full relaxation, considerable modifications of the surface structures for two components are observed due to the interaction between KTaO_3_ surface and NiO surface. As shown in Fig. 1(c), the equilibrium distances are 2.166 Å between the top-most Ta atom and the bottom-most O atom in NiO, and 2.183 Å between the O atom in TaO_2_-termination and the bottom-most Ni atom, respectively. The Ta atom of the top layer moves towards the NiO(001) surface, which results in the increase of the Ta–O bond length from 1.845 Å in pure KTaO_3_(001) surface to 1.895 Å in the interfacial structure. The O atom in the bottom layer of NiO side moves downwards and lengthens the distance between O and Ni from 2.157 Å in the pure surface to 2.417 Å in the interface.

The interface binding energy is calculated to evaluate the thermodynamic stability of NiO/KTaO_3_ heterostructure according to eqn (1):

1 *E*_ad_ = (*E*_KTaO_3_(001)_ + *E*_NiO(001)_ − *E*_interface_)/*A*

where *E*_interface_, *E*_KTaO_3_(001)_ and *E*_NiO(001)_ are the total energy of relaxed NiO(001)/KTaO_3_(001) interfacial structure, isolated KTaO_3_(001) and isolated NiO(001) slabs, respectively; *A* is the interfacial area of the slab. Based on the definition, a positive energy value means the interfacial structure is energetically stable and could be easily constructed. The computed binding energy is 0.39 eV Å^−2^ for the studied interface, which is comparable to the values of similar structures, such as YAlO_3_(001)/TiC(100) (0.36 eV Å^−2^),^73^ NiTi(111)/α-Al_2_O_3_(0001) (0.14 eV Å^−2^),^74^ and much more than typical van der Waals binding energy (13–21 meV Å^−2^).^75^ Therefore, the relaxed interfacial distances and calculated energies reveal that there is a covalent interaction in the interface between NiO(001) and KTaO_3_(001) surfaces.

### 3.2. Electronic structure

**Fig. 2:**
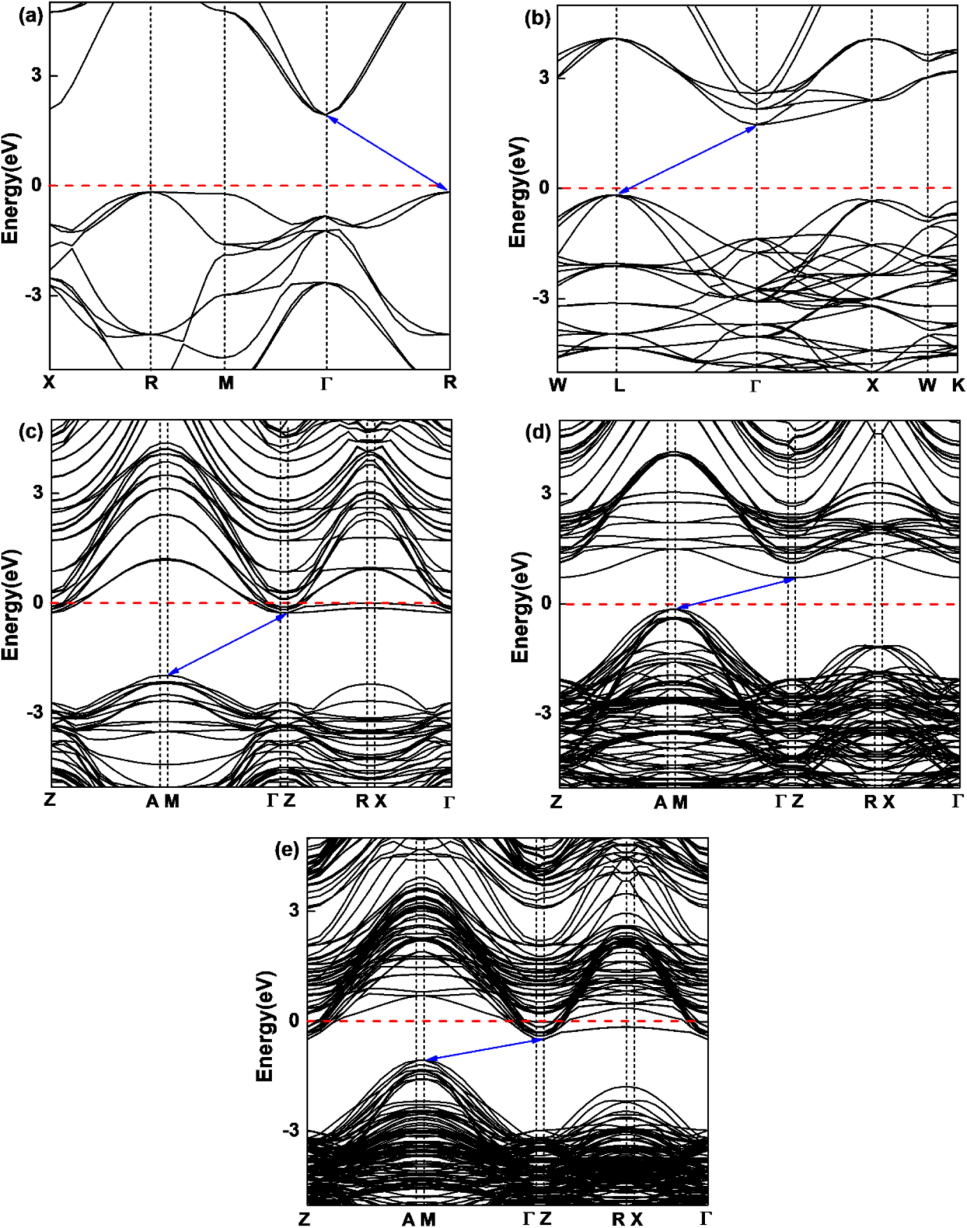
Calculated band structures of (a) bulk KTaO_3_ and (b) bulk NiO, (c) KTaO_3_(001), (d) NiO(001), and (e) NiO(001)/KTaO_3_(001). The Fermi level is set to be zero as a horizontal dashed red line.

In order to understand the influence of interfacial orbital hybridization on electronic structures, we have calculated band structures of bulk KTaO_3_, bulk NiO, KTaO_3_(001), NiO(001) and NiO(001)/KTaO_3_(001) heterostructure by means of PBE+U method. Fig. 2 summarizes the computed results drawn along high symmetry lines of the Brillouin zone. As shown in Fig. 2(a), an indirect band gap is predicted to be 2.10 eV for bulk KTaO_3_ with the valence band maximum (VBM) at the *R* point and the conduction band minimum (CBM) at the *Γ* point, which are consistent with previous DFT calculations.^76,77^Fig. 2(b) indicates that bulk NiO has an indirect band gap of 1.95 eV and the CBM and VBM are positioned at the *L* point and the *Γ* point, respectively. Although the calculated gap widths are smaller than experimental values, the dispersion features of bands are reasonable compared with previous theoretical findings by higher level calculations.^78^ As displayed in Fig. 2(c) and (d), the predicted band gaps are 1.70 eV for pure KTaO_3_(001) surface and 0.88 eV for pure NiO(001) surface, which are smaller than those of the corresponding bulks due to the existence of surface dangling bonds. The characterizations of indirect band gaps maintain in isolated surfaces with the VBM situated at the *M* point and the CBM located at the *Γ* point for both materials. The result in Fig. 2(e) demonstrates that NiO(001)/KTaO_3_(001) interface possesses an indirect band gap with the VBM located at the *M* point and the CBM positioned at the *Γ* point as well. The interfacial band gap of 0.67 eV is narrower than those of isolated surfaces. For isolated KTaO_3_(001) and NiO(001)/KTaO_3_(001) heterostructure, the Fermi levels cross the CBM, showing a n-type-like conductivity, which is mainly due to the surface charge redistribution.^46^ Under light illumination, the electrons in a semiconductor photocatalytic system are excited from the VBM to the CBM and the holes are generated in the VBM. An indirect band gap is favorable for restraining the recombination of photogenerated electron–hole pairs.^79^ Therefore, the calculated band structures reveal that the formation of NiO(001)/KTaO_3_(001) interface benefits the separation of photoinduced electrons and holes.

In the semiconductor-based photocatalysis, the separation efficiency and mobility of the photogenerated electrons and holes are very crucial for the reaction activity. Since the drift velocity of electrons or holes is inversely proportional to the effective mass, a lower effective mass would imply a higher mobility of charge carriers. In order to investigate the transfer properties of photogenerated carriers, the effective masses of electrons 
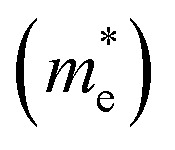
 and holes 
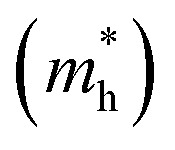
 of NiO(001)/KTaO_3_(001) and components are calculated by parabolic fitting to the CBM and VBM in Fig. 2(c)–(e) according to the following equation:^80,81^2
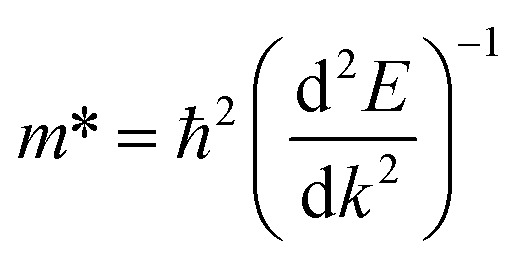
where m* is the effective mass of carriers, *ħ* is the reduced Planck constant and d^2^*E*/d*k*^2^ is the coefficient of the second-order term in a quadratic fit *E*(*k*) curves for the band edge. For KTaO_3_(001) surface, the electron and hole effective masses are 0.53 and 1.17, respectively, which indicates the transfer of electrons is faster than that of holes. For NiO(001), the calculated electron effective mass of 1.57 is larger than the hole effective mass of 0.66. After forming NiO(001)/KTaO_3_(001) interface, the band structure of KTaO_3_(001) is modified since the VBM is mainly composed of NiO states. As a result, the hole effective mass remarkably decreases to 0.58. The effective mass of electrons is 0.45, which is similar with that of pure KTaO_3_(001) because the CBM of the interfacial structure is mostly comprised of KTaO_3_ states. The results indicate the mobilities of photogenerated electrons and holes in the investigated interfacial structure are greater than those in each component due to the smaller effective masses of photoinduced carriers.

**Fig. 3:**
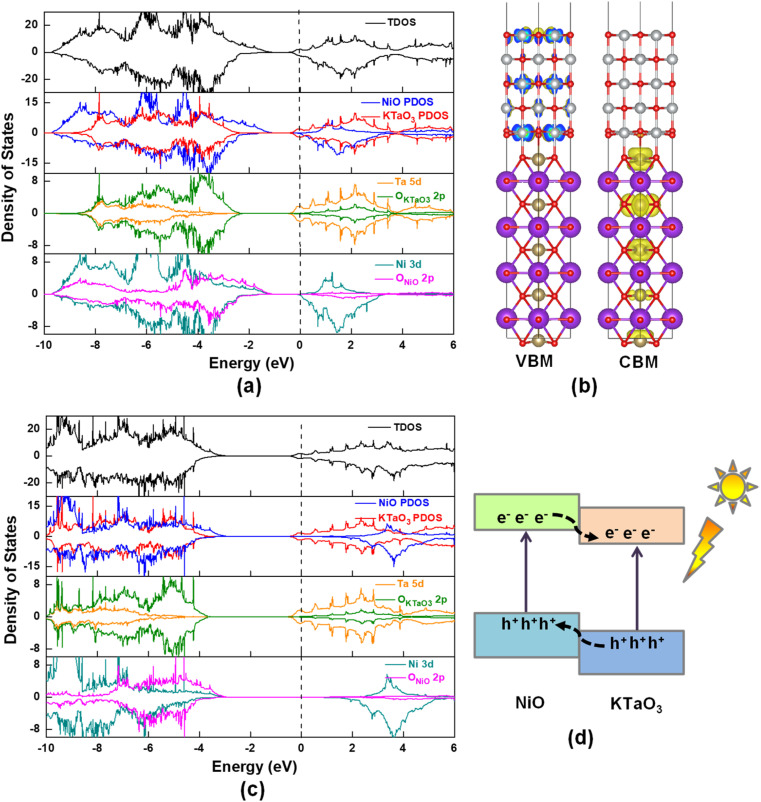
(a) Calculated TDOS and PDOS of NiO(001)/KTaO_3_(001) by using PBE+U method, (b) the charge density distributions of VBM and CBM obtained by VESTA, (c) calculated TDOS and PDOS by using HSE06 method, (d) the band alignment of two components in NiO(001)/KTaO_3_(001).

Fig. 3 displays total density of states (TDOS) and project density of states (PDOS) for NiO(001)/KTaO_3_(001) model. The Fermi level is represented by a vertical dashed line at zero. Fig. 3(a) suggests that there are no interfacial states in the forbidden gap calculated by PBE+U method. High quality interfaces will not trap the photogenerated carriers. The PDOS analyses show that the VBM is mainly from the contributions of NiO states and the CBM primarily consists of KTaO_3_ states, which are also described by the charge density distributions in Fig. 3(b). Further investigations on each component suggest that the VBM and CBM of KTaO_3_ slab in the interface are mostly comprised of O 2p states and Ta 5d states, respectively, which are similar with those in bulk.^41^ As to NiO side in the interface, the VBM is primarily characterized by the mixture of Ni 3d and O 2p states, while the CBM dominantly comes from Ni 3d states.

To evaluate the reasonability and reliability of PBE+U results, the hybrid functional HSE06 is applied to carry out the TDOS and PDOS analyses of NiO(001)/KTaO_3_(001). A comparison of results obtained by HSE06 and PBE+U methods demonstrates that, there is no apparent difference in the features of all the diagrams, except the width of band gaps. As shown in Fig. 3(c), the calculated band gap is 2.30 eV, remarkably larger than that obtained by PBE+U. The PDOS analyses indicate that the VBM has the main characteristics of NiO states and the CBM has the dominant characteristics of KTaO_3_ states, which are similar with the predictions by PBE+U. Since HSE06 calculations are much more time-consuming than PBE+U calculations, PBE+U method is utilized to the following calculations. It is shown in Fig. 3(d) that the energy levels of NiO states are higher than those of KTaO_3_ states in both the VBM and the CBM, indicating a typical type-II band alignment in NiO(001)/KTaO_3_(001).^82^ Under proper light irradiation, electrons will be excited to the CBM of semiconductor and accumulate on the KTaO_3_ side, while the photogenerated holes will transfer to NiO part. Our calculations support previous experimental findings that NiO serves as an oxidation cocatalyst is favorable for the separation and transfer of charge carriers.^35^

### 3.3. Charge density difference

**Fig. 4:**
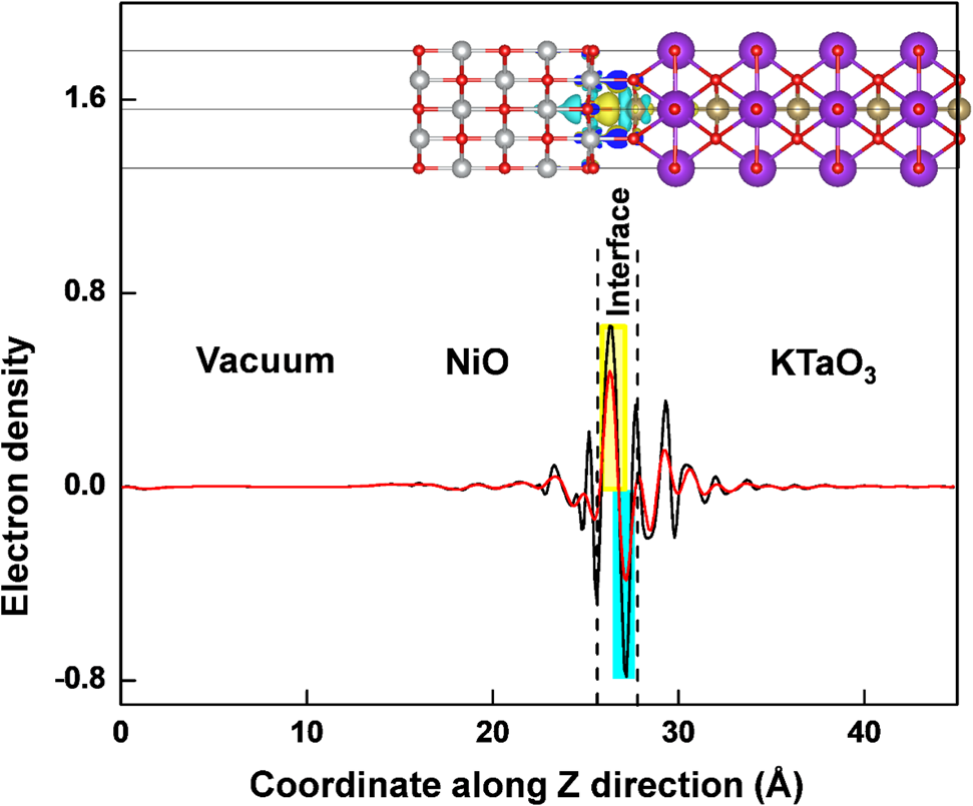
The three-dimensional charge density differences and the corresponding planar average differences (black line) and integral charge transfer (red line) for NiO(001)/KTaO_3_(001) interface. The yellow and cyan regions indicate electron accumulation and depletion, respectively. The isosurface value is 0.002 e A^−3^.

The interaction between KTaO_3_(001) surface and NiO(001) surface would lead to charge redistribution occurring at the interface. The interlayer interaction can be intuitively visualized from the three-dimensional charge density difference, Δ*ρ* = *ρ*_NiO/KTaO_3__ − *ρ*_KTaO_3__ − *ρ*_NiO_, where *ρ*_NiO/KTaO_3__, *ρ*_KTaO_3__, and *ρ*_NiO_ represent the respective charge densities of NiO(001)/KTaO_3_(001) interface, isolated KTaO_3_(001) surface and isolated NiO(001) surface. As shown in the upper part of Fig. 4, the yellow and cyan regions indicate electron accumulation and depletion, respectively. For the interface, charge rearrangements mainly occur between the top atoms of the KTaO_3_ surface and the bottom atoms of the NiO, and few contributions are observed from the atoms far away from the interface because of the weak interaction between inner atoms of individual surfaces. The electrons transfer from the Ta atoms to the O atoms in NiO surface, reflecting covalent bonding across the interface. Furthermore, the planar averaged charge density difference along the *Z* direction of the interfacial structure is computed and drawn in the lower part of Fig. 4. The positive and negative values indicate electron accumulation and depletion, respectively. It is shown that electrons accumulate in the NiO side and deplete from the KTaO_3_ side in the interfacial region, elucidating that there is a significant charge rearrangement occurring in the vicinity of the interface. A dipole pointing from KTaO_3_ to NiO should be generated, and the induced electric field is in favor of the separation of photogenerated carriers. Under the influence of this internal electric field, the photoinduced electrons migrate from NiO to KTaO_3_ and the photoinduced holes migrate from KTaO_3_ to NiO. Consequently, the photogenerated electrons and holes are spatially separated into two sides of NiO(001)/KTaO_3_(001) photocatalytic system and the charge recombination is restrained, which benefits the enhancement of photocatalytic activity.

### 3.4. Work function

**Fig. 5:**
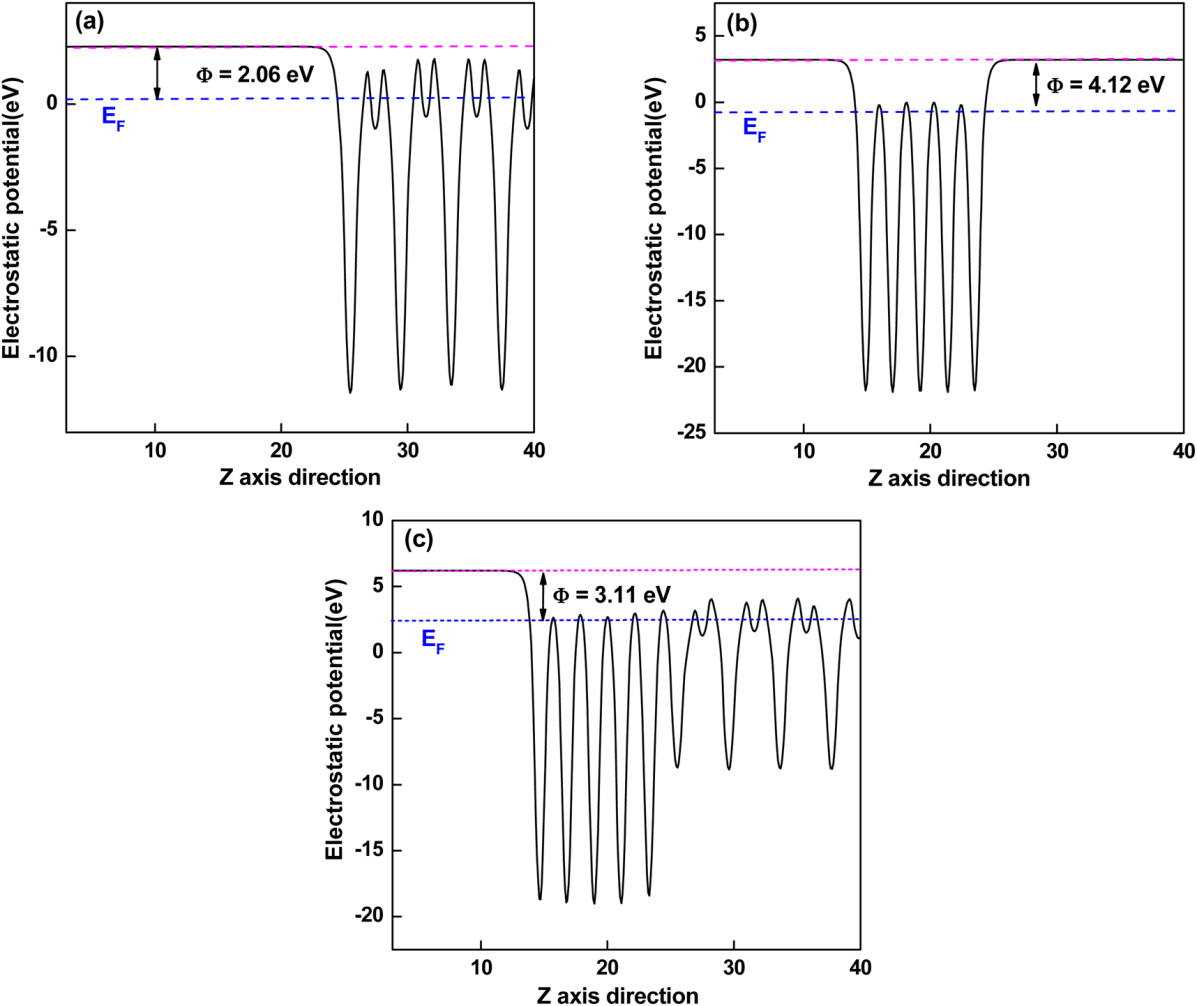
Calculated electrostatic potentials of (a) KTaO_3_(001), (b) NiO(001), and (c) NiO(001)/KTaO_3_(001) heterostructure. The magenta and blue dashed lines indicate the vacuum energy level and Fermi level, respectively.

The work function of a material is the minimum energy required to remove an electron from the bulk through the surface to the vacuum, which is an important parameter as an intrinsic reference for band alignment. Here, the work function (*Φ*) is defined as the difference between the potential energy of one electron between Fermi level and vacuum level according to eqn (3):

3 *Φ* = *V*_vac_ − *E*_F_

where *V*_vac_ is the electrostatic potential of the vacuum level and *E*_F_ is the Fermi energy. As shown in Fig. 5(a) and (b), the work function values of isolated KTaO_3_(001) surface and isolated NiO(001) surface are 2.06 eV and 4.12 eV, respectively. After forming NiO(001)/KTaO_3_(001) heterostructure, the electrons in KTaO_3_ with a lower work function flow into NiO with a higher work function, which is useful to the charge transfer and leading to a built-in potential at the interface. As a result, the NiO side of the interface gathers electrons, which is in good agreement with the analysis of charge density difference. The work function of NiO(001)/KTaO_3_(001) interface is equal to 3.11 eV shown in Fig. 5(c) owing to the strong interfacial charge transfer.

### 3.5. Optical property

**Fig. 6:**
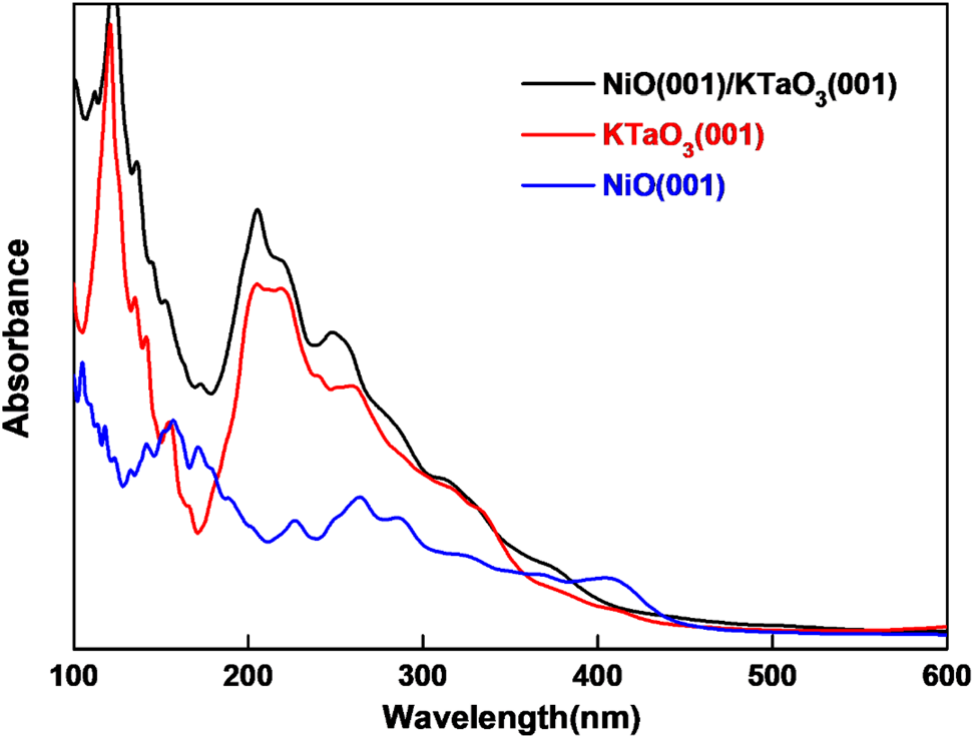
Computed optical absorption curves of NiO(001)/KTaO_3_(001), KTaO_3_(001) and NiO(001) slab models.

The optical absorption property of a photocatalytic material is a key parameter in describing the photocatalytic activity, which is highly related to its crystal and electronic structures.^83^ The photon energy dependent absorption coefficient *α*(*ω*) of the studied photocatalytic system is calculated according to eqn (4):^84,85^4
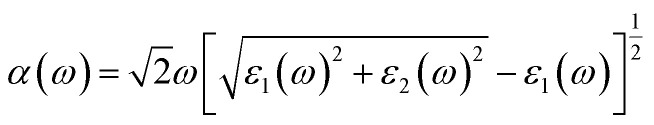
where *ε*_1_(*ω*) and *ε*_2_(*ω*) are the real and imaginary parts of dielectric function respectively. *ε*_2_(*ω*) is calculated in the random phase approximation, and *ε*_1_(*ω*) is generated from *ε*_2_(*ω*) by means of the Kramers–Kronig relation. The calculated absorption spectra of NiO(001)/KTaO_3_(001) interface, isolated KTaO_3_(001) surface and isolated NiO(001) surface are depicted in Fig. 6. It is found that the absorption spectrum of the interface has the similar character with that of isolated KTaO_3_(001) surface and stronger absorbance in the short wavelength region. There is no extension of absorption band edge observed, which indicates that the orbital hybridization and the electronic transitions between KTaO_3_(001) and NiO(001) are negligible. Our results demonstrate that the structural reconstruction around the interface plays a small role in extending the light absorption range, which is consistent with the experimental phenomenon of no change of band gap after loading NiO cocatalyst in KTaO_3_ photocatalyst.^39^

### 3.6. The mechanism of oxygen evolution reaction

The predicted results above demonstrate that NiO could act as an oxidation cocatalyst during the photocatalytic process. The oxygen evolution reaction (OER) by splitting water involves four-electron transfer, which is more complicated than the hydrogen evolution reaction and thus considered to be the rate-determining step in the whole reaction.^86^ In this work, we only investigate the thermodynamic process of OER using the approach proposed by Nørskov *et al.*^87,88^ In the electrochemical computation, OER generally includes four elementary steps at pH = 0, in which the electron transfer is coupled with proton removal as follows:

A H_2_O + * → HO* + H^+^ + e^−^

B HO* → O* + H^+^ + e^−^

C H_2_O + O* → HOO* + H^+^ + e^−^

D HOO* → * + O_2_ + H^+^ + e^−^

where * represents a surface of semiconductor, and HO*, O* and HOO* represent the adsorbed intermediates in OER. If the standard hydrogen electrode is taken as reference, the free energy of H^+^ + e^−^ is equal to be half the formation energy of H_2_ at the standard condition (pH = 0, *p* = 1 bar and *T* = 298 K). The Gibbs free energy change of each step at the standard condition is described by

5 Δ*G* = Δ*E* + ΔZPE − *T*Δ*S* − e*U*

where Δ*E* is the energy change of each step, the zero-point energy change ΔZPE and entropic contributions *T*Δ*S* are computed by using calculated vibrational frequencies and standard tables for the reactants and products in the gas phase.^88^ The entropies for the atoms and molecules adsorbed to the active site are assumed to be zero. Applying an external bias *U* on each proton-coupled electron transfer step is accounted by including a −e*U* term in the reaction free energy. The free-energy change of the total reaction 2H_2_O → O_2_ + 2H_2_ is fixed at the experimental value of 4.92 eV. For an ideal catalyst for OER, the four steps have the same reaction free energies of 1.23 eV. However, realistic photocatalysts do not show this behavior. Based on the Gibbs free energy changes of reactions (A)–(D), the overpotential of OER (*η*^OER^) can be computed by the following equation:6
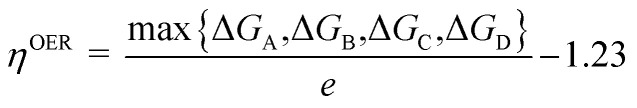
in which Δ*G*_A_, Δ*G*_B_, Δ*G*_C_, Δ*G*_D_ are the Gibbs free energy change of the elementary steps (A)–(D), respectively. According to the equation above, the lower the overpotential, the better the OER performance of a given photocatalyst.

**Fig. 7:**
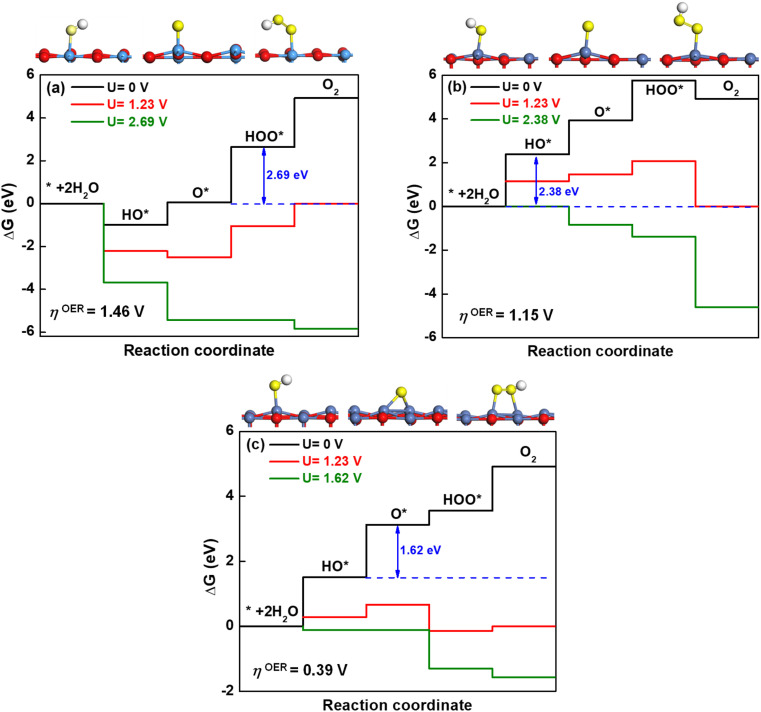
Free-energy diagram at pH = 0 and *T* = 298 K for the four steps of the OER at different applied potentials. The relaxed structures of intermediates are attached, in which the light blue, red, dark blue, yellow, and white balls represent the Ta, O in the surface, Ni, O in water, and H atoms, respectively.

The optimized geometries for the most stable oxidation species, including hydroxyl (HO*), oxygen (O*) and hydroperoxo (HOO*) are displayed in Fig. 7(a)–(c) for KTaO_3_(001), NiO(001) and NiO(001)/KTaO_3_(001), respectively. Based on these structures, the free-energy diagrams of OER at pH = 0, *T* = 298 K and different applied potentials are drawn in Fig. 7. At *U* = 0 V and the standard equilibrium potential for OER (*U* = 1.23 V), most of the steps are uphill. Therefore, it is necessary to add an overpotential on all the surfaces to make every step downhill. As to KTaO_3_(001), the calculated Δ*G*_HO*_ value for the first step is −0.99 eV, indicative of strong interaction between hydroxyl and the Ta atom in the surface. This strong binding character may easily poison the catalyst.^89^ Consequently, the third step to generate a HOO* intermediate becomes the potential-determining step. The corresponding overpotential is 1.46 V. For NiO(001), the computed value of Δ*G*_HO*_ for the first step is 2.38 eV. The binding strength of HO* is too weak, making the water difficult to dissociate into a HO* group in this step. As a result, the first step becomes the potential-determining step, requiring an overpotential of 1.15 V. For NiO(001)/KTaO_3_(001), the calculated Δ*G*_A_, Δ*G*_B_, Δ*G*_C_ and Δ*G*_D_ are 1.51, 1.62, 0.43 and 1.36 eV, respectively. Consequently, the second step determines the potential, in which OH* is deprotonated to O* with the O atom in water leaning to a near Ta atom in the surface and forming an extra Ta–O bond. The corresponding overpotential is equal to 0.39 V. As can be seen, the formation of the interfacial structure between NiO and KTaO_3_ results in the remarkable decrease of overpotential for OER and benefits the occurrence of photocatalytic water oxidation (Fig. 7).

## 4. Conclusion

We have performed DFT first-principles simulations to investigate the interfacial structures, electronic properties, optical absorption, charge transfer and band alignment of NiO/KTaO_3_ heterostructure. The binding energy and interfacial equilibrium distances indicate that there is a covalent interaction between KTaO_3_(001) and NiO(001). The analyses of band structure, charge density difference, and work function demonstrate that NiO/KTaO_3_ composite has an indirect band gap and an induced internal electric field at the interface, which are driving forces for the carrier migration to different regions of interface. It is found that both PBE+U and HSE06 produce a type-II band alignment for NiO(001)/KTaO_3_(001) slab model, with the accumulation of photogenerated electrons on KTaO_3_ side and the gathering of photoinduced holes on NiO side. These characteristics benefit the separation of photogenerated electrons and holes and increase the lifetime of carrier charges. The interfacial formation has a small impact on the optical absorption of photocatalytic system, which is in good agreement with experimental results. The addition of NiO layer on KTaO_3_ surface makes the adsorption of water oxidation species more moderate, which importantly decreases the overpotential of OER. This work reveals an important role of NiO as the oxidation cocatalyst to separate the charge carriers in KTaO_3_ photocatalytic system and provides an insight into the mechanism of the remarkable enhancement of photocatalytic activity in KTaO_3_ by loading NiO particles.

## Conflicts of interest

There are no conflicts to declare.

## Supplementary Material
